# Improving global influenza surveillance: trends of A(H5N1) virus in Africa and Asia

**DOI:** 10.1186/1756-0500-5-62

**Published:** 2012-01-23

**Authors:** Magdalena Escorcia, Matias S Attene-Ramos, Marco Juarez Estrada, Gerardo M Nava

**Affiliations:** 1 Departamento de Medicina y Zootecnia de Aves. Facultad de Medicina Veterinaria y Zootecnia, Universidad Nacional Autónoma de México. Ciudad Universitaria, Coyoacán, DF 04510, Mexico; 2 NIH Chemical Genomics Center, National Human Genome Research Institute, NIH, 9800 Medical Center Drive, Rockville, MD 20850, USA; 3 Dept. Pathology and Immunology, Washington University School of Medicine, Box 8118, 660 S. Euclid Ave, St. Louis, MO 63110, USA

## Abstract

**Background:**

Highly pathogenic avian influenza A(H5N1) viruses are an important health problem in many Asian and African countries. The current increase in human cases demonstrates that influenza A(H5N1) is still a significant global pandemic threat. Many health organizations have recognized the need for new strategies to improve influenza global surveillance. Specifically, the World Health Organization through the global technical consultation for influenza surveillance have called for a detailed picture of the current limitations, especially at the nation level, to evaluate, standardize and strength reporting systems. The main goal of our study is to demonstrate the value of genetic surveillance as part of a strategic surveillance plan. As a proof of concept, we evaluated the current situation of influenza A(H5N1) in Asian and Africa.

**Results:**

Our analysis revealed a power-law distribution in the number of sequences of A(H5N1) viruses analyzed and/or reported to influenza surveillance networks. The majority of the Asian and African countries at great risk of A(H5N1) infections have very few (approximately three orders of magnitude) sequenced A(H5N1) viruses (e.g. hemagglutinin genes). This suggests that countries under pandemic alert for avian influenza A(H5N1) have very limited participation (e.g. data generation, genetic analysis and data share) in avian influenza A(H5N1) surveillance. More important, this study demonstrates the usefulness of influenza genetic surveillance to detect emerging pandemic threat viruses.

**Conclusions:**

Our study reveals that some countries suffering from human cases of avian influenza have limited participation (e.g. genetic surveillance or data share) with global surveillance networks. Also, we demonstrate that the implementation of genetic surveillance programs could increase and strengthen worldwide epidemic and pandemic preparedness. We hope that this work promotes new discussions between policy makers and health surveillance organizations to improve current methodologies and regulations.

## Findings

The recent outbreaks of highly pathogenic avian influenza A(H5N1) virus in numerous countries in Asia and Africa and the increase in human cases, demonstrate that influenza A viruses remain a global pandemic threat [1,2]. Worldwide, natural migrations of birds and commercialization of poultry product are considered two of the most important mechanisms of disease dispersion [3]. Due to the high risk of the A(H5N1) pandemic threat, multinational efforts have been made to improve the surveillance and control of avian influenza viruses around the world [4]. In recent years, important progress has been accomplished in the standardization of laboratory techniques for the diagnostics of influenza viruses [5], and phylogenetic nomenclature of highly pathogenic avian influenza A(H5N1) virus [6]. These efforts have generated establishment of advanced global networks for influenza surveillance [7].

In response to the 2009 influenza pandemic threat, the World Health Organization (WHO) through the global technical consultation for influenza surveillance has called for a detailed picture of current limitations in national reporting systems [8,9]. An important component of the influenza surveillance system that deserves significant attention is the monitoring of genetic and antigenic changes occurring in influenza viruses that circulate among human and animals (e.g. [10,11]).

Genetic surveillance of influenza viruses have shown to be an essential tool for establishing the gene origin of pandemic viruses, monitoring the status of an outbreak [11,12] and design control strategies [13,14]. However, the regular use of genetic influenza surveillance remains limited to only a few countries. Herein, we evaluate the extent of this problem and demonstrate the importance of genetic surveillance during the current situation of highly pathogenic avian influenza A(H5N1) virus in Asia and Africa. Given the significance of this global pandemic threat, this study provides some basis to improve our current surveillance programs.

### Surveillance of avian influenza H5N1 virus in Asia and Africa

Information of human cases of avian influenza A(H5N1) reported to the WHO, were retrieved from the Global Alert and Response (GAR) system [15]. This information has been collected under WHO policies and regulations, and includes epidemiological data and operational information about disease outbreaks. This system manages critical information about outbreaks and accurate and timely communications between international public health professionals [16]. A written consent (WHP-73,056) to extract and publish the data (number human cases) was obtained from the WHO.

Avian influenza A(H5N1) have been reported in 15 countries (as of 25th March, 2011): Azerbaijan, Bangladesh, Cambodia, China, Djibouti, Egypt, Indonesia, Iraq, Lao People's Democratic Republic, Myanmar, Nigeria, Pakistan, Thailand, Turkey and Viet Nam [15]. Because other countries in Asia, Africa and Europe are at prominent risk of influenza outbreaks, the global network for influenza surveillance has implemented specific surveillance polices for this region [7]. To gain insights into the current status and capacity of influenza A(H5N1) surveillance in these countries, we analyzed the number of A(H5N1) hemagglutinin (HA) sequences generated in national disease centers and universities as an indicator of active surveillance. Analysis of influenza HA proteins is one of the most important tools for global influenza surveillance network [2,17].

**Figure 1:**
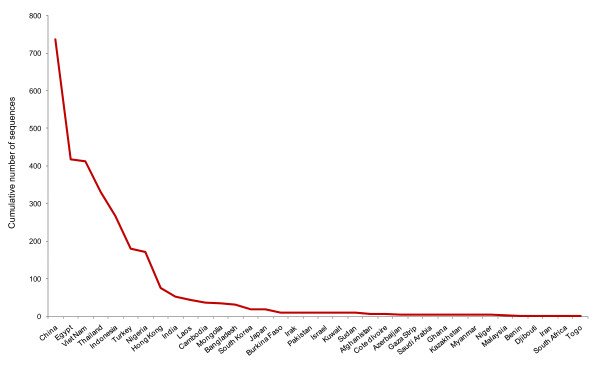
**Cumulative number of A(H5N1) hemagglutinin genes sequenced in Asian and African countries and reported to influenza surveillance networks between 2003 and 2011 approximates a power law distribution (nonlinear power law regression, R^2 ^= 0.9066 and Kolmogorov-Smirnov test *P <*0.0001 for lower, middle, and upper quartiles)**.

First, we examined the number of deposited HA sequences in the Influenza Virus Resource, a comprehensive database integrating data from the National Institute of Allergy and Infectious Diseases (NIAID), the J. Craig Venter Institute (JCVI) and GenBank at the National Center for Biotechnology Information (NCBI) [18]. We retrieved and archived records for HA sequences in Asian and African countries (all sequences available from different hosts and environmental samples) of influenza A virus subtype H5N1 from 2003 to 2011 (as of 25th March, 2011). A total of 2,934 HA sequences were found and retrieved from the database. We found that the cumulative number of sequences for this period of time (2003-2011) varied drastically between countries (Figure 1). The analysis revealed an approximation to a power law distribution showing some degree of inequality between nations. For example, most of the A(H5N1) sequences (73.7%) were obtained from a few number of countries (China, Egypt, Vietnam, Thailand and Indonesia) and of the 36 Asian and African countries that have performed genetic surveillance of HA proteins, 50% of them have deposited less than 10 sequences in the database between 2003 and 2011 (Additional file 1).

**Figure 2:**
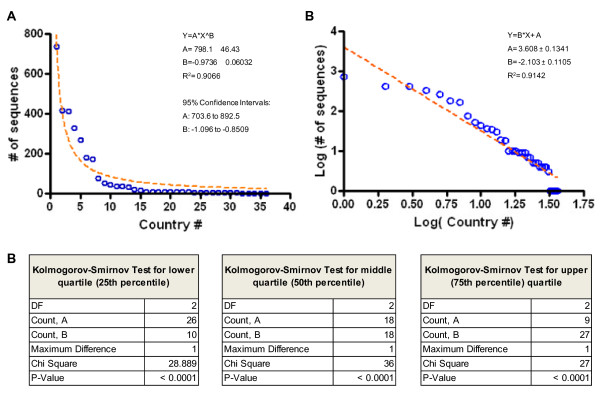
**Power law distributions of the cumulative number of A(H5N1) hemagglutinin genes sequenced in Asian and African countries and reported to influenza surveillance networks between 2003 and 2011 (data shown in Figure 1) The 36 countries were ranked by the number of reported gene sequences to the influenza surveillance network and plotted against the total number of sequences**. A) The data was fit to a nonlinear power law regression and the coefficients were estimated. B) Log-Log plot (same dataset) showing a linear regression. C) Kolmogorov-Smirnov test for goodness-of-fit to power law distributions derived from data variability using the lower, middle (median), and upper quartiles.

To verify the power law approximation in influenza genetic surveillance, we performed two independent statistical tests. First, we ranked the countries by the number of available sequences and then we fit the data to a nonlinear power law regression and a log-log plot using GraphPad Prism 4 software, (GraphPad Prism, CA. USA). Second, we performed the Kolmogorov-Smirnov test for goodness-of-fit to power law distributions [19] derived from data variability using the lower (25th percentile), middle (50th percentile or median), and upper (75th percentile) quartiles. This statistical analysis was carried out using StatView software version 5.0.1. (SAS Institute, NC. USA). These analyses confirmed that HA gene sequences archived in the in the Influenza Virus Resource have an approximation to a power law distribution (nonlinear power law regression, R^2 ^= 0.9066 and Kolmogorov-Smirnov test *P *< 0.0001 for lower, middle, and upper quartiles; Figure 2) demonstrating a strong sequencing disproportion between nations. Together, these data suggest that some of the selected nations have limited participation (e.g. data generation, genetic analysis and data share) in the genetic surveillance of avian influenza A(H5N1) or a limited interaction with global surveillance networks for data share. These observations highlight the need of new polices to improve collaboration between nations affected by influenza outbreaks.

**Figure 3:**
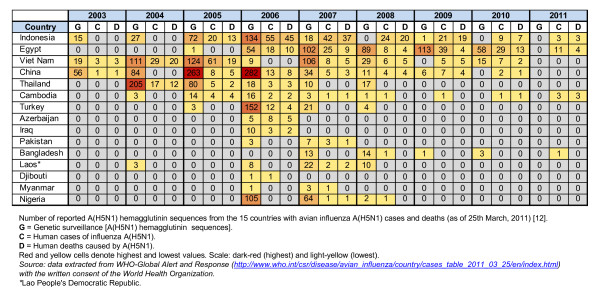
**Trends of influenza genetic surveillance in countries under current avian influenza A(H5N1) outbreaks in humans (disease cases and deaths)**. Number of reported A(H5N1) hemagglutinin sequences from the 15 countries with avian influenza A(H5N1) cases and deaths (as of 25th March, 2011) [12]. G = Genetic surveillance [A(H5N1) hemagglutinin sequences]. C = Human cases of influenza A(H5N1). D = Human deaths caused by A(H5N1). Red and yellow cells denote highest and lowest values. Scale: dark-red (highest) and light-yellow (lowest). Source: data extracted from WHO-Global Alert and Response http://www.who.int/csr/disease/avian_influenza/country/cases_table_2011_03_25/en/index.html with the written consent of the World Health Organization. *Lao People's Democratic Republic.

Second, to evaluate the trends of influenza genetic surveillance in countries under current avian influenza A(H5N1) outbreaks in humans, we examined the number of reported HA sequences from the 15 countries with avian influenza A(H5N1) disease cases and deaths (as of 25th March, 2011) [15]. Our analysis demonstrated that between the years 2003 and 2011, nations such as China, Egypt, Indonesia, Thailand and Viet Nam have performed a constant A(H5N1) genetic surveillance (Figure 3). Intriguingly, we observed instances of lack of A(H5N1) genetic surveillance even when human cases and deaths took place (e.g. Indonesia 2008, 2010, 2011 and Egypt 2011; Viet Nam 2009, Cambodia 2009, 2010, 2011). These results indicated that in many instances, genetic analysis of influenza A(H5N1) is used as a Ad hoc strategy rather than a surveillance method. Moreover, our results also demonstrate that genetic surveillance of influenza viruses is performed by disease control centers and research institutions with a limited number of sequences per year (e.g. Cambodia, 37 HA sequences in 8 year period).

**Figure 4:**
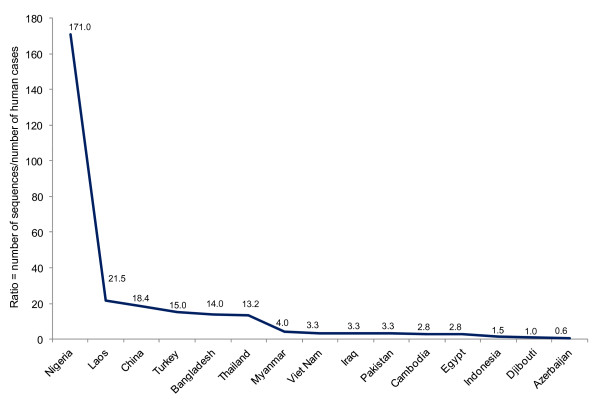
**Ratios between total number of avian influenza A(H5N1) HA sequences and the total number of human cases of influenza A(H5N1) (data between 2003 and 2011 as shown in Figure 3)**. This analysis revealed significant differences between countries. The ratio difference between Nigeria and Azerbaijan represent a 285 fold difference. Statistical analysis was performed by means of Kolmogorov-Smirnov test for goodness-of-fit to power law distributions using lower (*P *= 0.0025), middle (*P *= 0.0011), and upper (*P *= 0.0165) quartiles.

To demonstrate that some countries suffering from human cases of avian influenza have limited participation (i.e. genetic surveillance or data share) with global surveillance networks, we estimated the ratios between total number of avian influenza A(H5N1) HA sequences and total number of human cases of influenza A(H5N1) (data collected between 2003 and 2011, as shown in Figure 3). This analysis revealed significant differences (*P *≤ 0.016) in sequencing capability between countries (Figure 4). For example, in Nigeria 171 A(H5N2) genes were sequenced per human case; in contrast, in Azerbaijan only 0.6 genes were sequenced per human case. These numbers represent a 285 fold difference between these two countries. Together, these findings also indicate that some nations have limited participation in the genetic surveillance of avian influenza A(H5N1) or a limited interaction with global surveillance networks for data share. This problem was recently recognized by the WHO global technical consultation for influenza surveillance. This group of experts identify that the Ad hoc surveillance strategy is easy to use but is least desirable because results are biased depending on sampling approach [8]. Together, these observations reaffirm the need of new policies in influenza surveillance to improve country coverage and sampling prioritization.

We recognize that our assessment has been restricted to the genetic surveillance of A(H5N1) viruses and data deposition in influenza global networks, and that serological surveillance may occur in some of these countries. Nevertheless, the main goal of our analysis is to illustrate the current situation of some nations facing the risk of human influenza outbreaks and their involvement with the global influenza surveillance network.

These observations emphasize the need of new polices to improve genetic surveillance and data share for influenza surveillance. For example, a better distribution of financial and technical sources among affected nations. To demonstrate the importance of implementing genetic analysis and surveillance networks, we performed a retrospective and comprehensive genetic analysis of influenza A(H5N1) viruses in these 15 Asia and Africa countries. We analyzed A(H5N1) genetic diversity between years 2000 and 2011. A total of 2,757 sequences (as of 25th March, 2011) were retrieved from the Influenza Virus Resource at the NCBI [18]. After removing duplicated and partial protein sequences, 1,446 HA sequences were aligned using MUSCLE software [20], manually inspected for quality and trimmed to equal length. Aligned sequences were used for genetic analysis using the minimum evolution method [21]. Analysis were performed using the MEGA4 [22] and Seaview [23] software. The statistical significance of branch order was estimated by the generation of 50 replications of bootstrap re-sampling of the originally-aligned nucleotide sequences.

**Figure 5:**
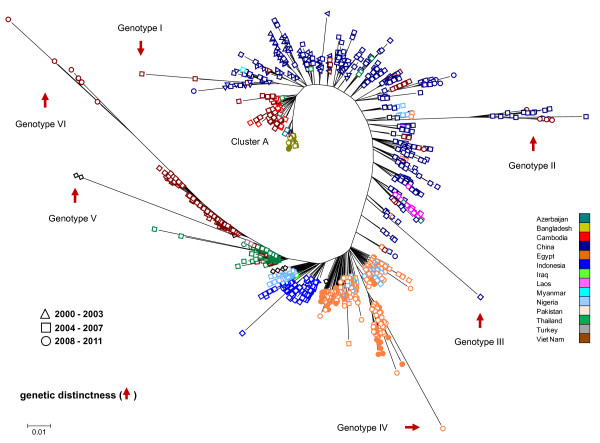
**The genetic diversity and distinctiveness of influenza A(H5N1) viruses circulating between 2003 and 2011 in Asian and African countries**. Filled circles depict viruses isolated in 2010-2011.

The genetic analysis of HA proteins from influenza A(H5N1) viruses circulating between 2000 and 2011 in the selected Asian and African countries, revealed that A(H5N1) viruses clustered by country and year of isolation indicating that this virus follows regional and annual trends (Figure 5). Also, the analysis revealed that viruses circulating in China are highly diverse, supporting the idea that this region represents an important source of highly diverse pathogenic H5N1 avian influenza viruses [24]. For example, viruses circulating in China between 2000 and 2003 are genetic similarity to those circulating in Cambodia, Vietnam and Laos in the period between 2004 and 2007, and in Bangladesh between 2007 and 2010 (e.g. Figure 5, cluster A). In many countries, recent (2008-2011) A(H5N1) viral isolates have close homology to viruses circulating in the period between 2004 and 2007 (e.g. Figure 5; genotype II). More important, our genetic surveillance method was able to identify A(H5N1) viruses with genetic distinctness, an important trait of novel and potentially pandemic influenza viruses [12]. Together, these results highlight the value of genetic surveillance to accurately indentify genetic differences between influenza viruses that circulate among humans and animals, a key factor for the development of vaccines, outbreak preparedness, and pandemic planning (e.g. [10-13,25,26]).

To corroborate our observations, we examined the origin of the viruses with genetic distinctiveness. Genotype I (e.g. Viet Nam 2005, accession numbers ABD14806 and ABD14808) includes highly pathogenic viruses isolated from shell washes of duck and goose eggs confiscated from travelers. These viruses have close homology to a Hong Kong 97-like H5N1 influenza virus [27]. Genotype II (e.g. Viet Nam isolates from 2008 to 2011, ACO07034, ACO07035, ACO07036 and ACO07037) includes highly pathogenicity and exotic H5N1 viruses isolated from poultry seized at a port of entry into Viet Nam [28]. Genotype III (e.g. China 2006, ADG59089), an exotic virus generated through multiple reassortment events isolated in China. Importantly, viruses with close HA protein homology to genotype III, reacted poorly with antisera produced from other viruses circulating in China. Based on these observations, these viruses were cataloged as a pandemic threat [29]. Genotype IV (e.g. Egypt 2008, ADG28679; and 2010, ADM85867, ADM85879) includes recent isolates of highly pathogenicity avian influenza A(H5N1) viruses circulating in Egypt. Genotype V (USA 2007, ACZ36900 and ACZ48494) represents A(H5N1) sequences used to validate the efficacy of this genetic analysis to detect exotic genotypes. Genotype VI (e.g. Viet Nam 2008, ACY06607, ACY06606, ACO07033 and ADF83652) integrated by high pathogenicity A(H5N1) viruses isolated from chickens seized at ports of entry in Viet Nam [30]. These viruses were antigenically distinct from contemporary strains circulating in Viet Nam. This genotypic group also included A(H5N1) viruses isolated from humans possessing unknown virulence determinants involved in high pathogenicity [31]. Together, these analyses reveal the value of genetic surveillance to identify potential influenza pandemic threats circulating in specific global regions.

The influenza pandemic in 2009 highlighted the need of an improved global surveillance system. Specifically, the last pandemic event showed the technical challenges that many nations face to monitor influenza outbreaks when standardized surveillance and reporting system are not established [8]. Significant efforts have been taken by independent research groups and the WHO to revise and standardize methodologies for data collection and reporting systems. However, these challenges are very complex in nature and many of them remain ambiguous. For example, the WHO global technical consultation for influenza surveillance and other WHO influenza program experts recently indentify that the lack of standardization in surveillance methods results in data sets that are not comparable between nations and that the data cannot be compiled to generate a comprehensive picture of the virus spread between nations [8]. To this end, our study clearly documents this issue. Here, we have shown that many countries where highly pathogenic avian influenza A(H5N1) virus represents a current pandemic threat, have a limited participation (e.g. data generation, genetic analysis and data share) in avian influenza A(H5N1) surveillance. Although we cannot provide conclusive evidence explaining these national trends, we presume that this issue could be linked to the fact that some nations lack technical sources to perform the surveillance, lack an effective mechanism for reporting surveillance data, have limited human sources (trained personnel, problems with internet connectivity, etc.) or are influenced by political pressures [8].

Importantly, the WHO global technical consultation for influenza surveillance has also called for the description of a detailed picture of the current limitations, especially at the nation level [8,9]. With this in mind, here we provide some bases to establish a strategy to overcome some of these limitations and enhance influenza surveillance. Specifically, our analysis demonstrates that implementation of genetic surveillance programs could strengthen worldwide epidemic and pandemic preparedness. For example, our study revealed that A(H5N1) viruses clustered by country and year of isolation indicating that this virus follows regional and annual trends. Also, our analysis identified highly diverse (exotic) viruses co-circulating with endemic viruses. Based on these results, we propose that genetic surveillance system should be a key component of influenza surveillance. In particular, we believe that genetic surveillance can be adopted worldwide even in less-resourced areas. This idea is supported by studies demonstrating that a few genetic sequences (less than 50 HA genes sequenced) are sufficient to effectively track the antigentic changes occurring in influenza viruses circulating in specific geographical locations and over a period of 5-10 years [13,25]. We hope the present study stimulates further studies and discussions to accelerate the improvement of guidance and recommendations for influenza surveillance.

In summary, the significance of the present study is at least three fold. First, it is revealed that in many countries under current pandemic alert for avian influenza A(H5N1), there is a limited genetic surveillance of influenza A(H5N1) viruses. This is due apparently to a limited participation (e.g. data generation, genetic analysis and data share) in avian influenza A(H5N1) surveillance. Second, analysis of national surveillance trends revealed that genetic analysis of influenza viruses occurred in many cases as Ad hoc strategy but not as a surveillance technique (prevention). Third, it is demonstrated the usefulness of influenza genetic surveillance to detect pandemic threat viruses. The significance of this molecular approach was demonstrated in the past A(H1N1) 2009 world pandemic, in which genetic analyses were essential to establish the origin of the new reassortant virus [11,12]. Finally, and perhaps more important, it is worrying that the majority of countries suffering from human cases of avian influenza have a limited access to genetic surveillance, even though this method is very effective to detect exotic and highly divergent circulating between humans, wild and farm animals. We wish that this work promotes new discussions between policy makers and health surveillance organizations to improve current methodologies and regulations.

## Abbreviations

WHO: World Health Organization; GAR: Global Alert and Response; HA: Hemagglutinin; NIAID: National Institute of Allergy and Infectious Diseases; JCVI: The J. Craig Venter Institute; NCBI: The National Center for Biotechnology Information.

## Competing interests

The authors declare that they have no competing interests.

## Authors' contributions

GMN, ME, MJE and MSAR designed research and performed research; GMN, ME and MSAR wrote the paper. All authors read and approved the final manuscript.

## Supplementary Material

Additional file 1 **Additional table**. Cumulative number of A(H5N1) hemagglutinin genes sequenced in Asian and African countries and reported to influenza surveillance networks between 2003 and 2011. Click here for file
