# Supplementary material for: Improving global influenza surveillance: trends of A(H5N1) virus in Africa and Asia
Source: BMC Res Notes. 2012 Jan 23;5:62. doi: 10.1186/1756-0500-5-62 (PMC3305413; doi:10.1186/1756-0500-5-62)
Supplement: Additional file 1 — Additional table. Cumulative number of A(H5N1) hemagglutinin genes sequenced in Asian and African countries and reported to influenza surveillance networks between 2003 and 2011. [file 1756-0500-5-62-S1.PDF]

Additional File 1. Additional table. Cumulative number of A(H5N1) hemagglutinin genes sequenced in Asian and African countries and reported to influenza surveillance networks between 2003 and 2011.

	2003	2004	2005	2006	2007	2008	2009	2010	2011	Total
China	56	84	263	282	34	11	6	0	0	**736**
Egypt	0	0	1	54	102	89	113	58	0	**417**
Viet Nam	19	111	124	9	106	29	0	15	0	**413**
Thailand	0	205	80	18	10	17	0	0	0	**330**
Indonesia	15	27	72	134	18	0	1	0	0	**267**
Turkey	0	0	3	152	21	4	0	0	0	**180**
Nigeria	0	0	0	105	64	2	0	0	0	**171**
Hong Kong	12	3	1	18	27	7	6	1	0	**75**
India	0	0	0	8	2	32	8	2	0	**52**
Laos	0	3	0	8	22	10	0	0	0	**43**
Cambodia	0	3	14	16	3	1	0	0	0	**37**
Mongolia	0	0	6	2	0	0	23	4	0	**35**
Bangladesh	0	0	0	0	13	14	1	3	0	**31**
South Korea	3	1	0	9	0	6	0	0	0	**19**
Japan	1	7	0	0	1	5	1	0	3	**18**
Burkina Faso	0	0	0	10	0	0	0	0	0	**10**
Irak	0	0	0	10	0	0	0	0	0	**10**
Pakistan	0	0	0	3	7	0	0	0	0	**10**
Israel	0	0	0	6	0	1	0	2	0	**9**
Kuwait	0	0	0	0	9	0	0	0	0	**9**
Sudan	0	0	0	9	0	0	0	0	0	**9**
Afghanistan	0	0	0	7	0	0	0	0	0	**7**
Cote dIvoire	0	0	0	7	0	0	0	0	0	**7**
Azerbaijan	0	0	0	5	0	0	0	0	0	**5**
Gaza Strip	0	0	0	5	0	0	0	0	0	**5**
Saudi Arabia	0	0	1	0	4	0	0	0	0	**5**
Ghana	0	0	0	0	4	0	0	0	0	**4**
Kazakhstan	0	0	2	2	0	0	0	0	0	**4**
Myanmar	0	0	0	1	3	0	0	0	0	**4**
Niger	0	0	0	4	0	0	0	0	0	**4**
Malaysia	0	2	0	0	1	0	0	0	0	**3**
Benin	0	0	0	0	1	0	0	0	0	**1**
Djibouti	0	0	0	1	0	0	0	0	0	**1**
Iran	0	0	0	0	0	1	0	0	0	**1**
South Africa	0	1	0	0	0	0	0	0	0	**1**
Togo	0	0	0	0	1	0	0	0	0	**1**
**Total**	**106**	**447**	**567**	**885**	**453**	**229**	**159**	**85**	**3**	**2934**

Number of A(H5N1) hemagglutinin sequences deposited in the Influenza Virus Resource, a comprehensive database integrating data from the National Institute of Allergy and Infectious Diseases (NIAID), the J. Craig Venter Institute (JCVI) and GenBank at the National Center for Biotechnology Information [14]. (See text for details).
